# Anti-β-sheet conformation monoclonal antibody reduces tau and Aβ oligomer pathology in an Alzheimer’s disease model

**DOI:** 10.1186/s13195-018-0337-3

**Published:** 2018-01-29

**Authors:** Fernando Goñi, Mitchell Martá-Ariza, Krystal Herline, Daniel Peyser, Allal Boutajangout, Pankaj Mehta, Eleanor Drummond, Frances Prelli, Thomas Wisniewski

**Affiliations:** 1 Center for Cognitive Neurology and Department of Neurology, New York University School of Medicine, Alexandria, ERSP Rm 802, 450 East 29th Street, New York, NY USA; 2 Department of Pathology, New York University School of Medicine, New York, NY USA; 3 Department of Psychiatry, New York University School of Medicine, New York, NY USA; 4 Department of Immunology, New York State Institute for Basic Research in Developmental Disabilities, Staten Island, NY USA

**Keywords:** Immunomodulation, Oligomers, Amyloid-β, Tau, Prion

## Abstract

**Background:**

Oligomeric forms of amyloid-β (Aβ) and tau are increasing being recognized as key toxins in the pathogenesis of Alzheimer’s disease (AD).

**Methods:**

We developed a novel monoclonal antibody (mAb), GW-23B7, that recognizes β-sheet secondary structure on pathological oligomers of neurodegenerative diseases.

**Results:**

The pentameric immunoglobulin M kappa chain (IgMκp) we developed specifically distinguishes intra- and extracellular pathology in human AD brains. Purified GW-23B7 showed a dissociation constant in the nanomolar range for oligomeric Aβ and did not bind monomeric Aβ. In enzyme-linked immunosorbent assays, it recognized oligomeric forms of both Aβ and hyperphosphorylated tau. Aged triple-transgenic AD mice with both Aβ and tau pathology infused intraperitoneally for 2 months showed IgMκp in the soluble brain homogenate, peaking at 24 h postinoculation. Treated mice exhibited significant cognitive rescue on radial arm maze testing compared with vehicle control-infused mice. Immunohistochemically, treatment resulted in a significant decrease of extracellular pathology. Biochemically, treatment resulted in significant reductions of oligomeric forms of Aβ and tau.

**Conclusions:**

These results suggest that GW-23B7, an anti-β-sheet conformational mAb humanized for clinical trials, may be an effective therapeutic agent for human AD.

**Electronic supplementary material:**

The online version of this article (10.1186/s13195-018-0337-3) contains supplementary material, which is available to authorized users.

## Background

The pathogenesis of many neurodegenerative diseases (NDDs) involves misfolding of a protein or peptide from a soluble physiological conformer to a pathological oligomeric form that can spread in a prion-like manner before contributing to insoluble fibrillar deposits [1–8]. Synaptic and cellular toxicity is associated mainly with the misfolded oligomeric conformers. The most common NDD, Alzheimer’s disease (AD), is characterized by two different misfolded proteins: the extracellular amyloid-β (Aβ) and the intracellular hyperphosphorylated tau (p-tau). Whereas the Aβ deposits form neuritic plaques and congophilic amyloid angiopathy, p-tau forms neurofibrillary tangles (NFTs) [9, 10]. Accumulation of Aβ pathology is thought to be a prerequisite for subsequent tau-related pathology in AD. Currently, there is no effective disease-modifying pharmacological or immunological therapeutic approach for AD, with current medications providing only limited symptomatic relief.

Nevertheless, there are a number of ongoing clinical trials in which researchers are using approaches with monoclonal antibodies (mAbs) or active vaccination that address the tau or Aβ pathology individually [11–13]. The majority of these approaches target Aβ-related pathology, several of which are in ongoing phase III clinical trials. So far, there has been a very high failure rate of AD trials targeting Aβ pathology, with more recent attempts addressing tau pathology, which correlates better with the cognitive deficits in AD [11–15]. We believe that addressing both of these pathologies concurrently might be a more optimal approach; however, until recently, this has not been feasible [16, 17].

Previously, we demonstrated that immunizing mice with a synthetic non-self soluble antigen with a repetitive β-sheet secondary structure could induce sufficient numbers of B cells, which specifically recognize the defined secondary structure, to produce stable hybridomas by a selection process using pathological oligomeric forms of misfolded proteins/peptides of diverse NDDs [17]. One of the anti-β-sheet conformational monoclonal antibodies (aβComAb) generated, GW-23B7, showed affinity for both Aβ and tau oligomeric forms derived from human AD tissue. In the present study, we demonstrate that this novel aβComAb, GW-23B7, infused intraperitoneally (i.p.) could access the brains of triple-transgenic (3 × Tg) AD mice with both Aβ and tau pathologies and induce a significant cognitive rescue by reducing pathological oligomeric conformers of both tau and Aβ.

## Methods

### Production and selection of the conformational monoclonal antibody GW-23B7

The aβComAb GW-23B7 was obtained after immunization of a CD-1 mouse with the p13Bri immunogen and subsequent hybridoma production performed at the bi-institutional Antibody and Bioresource Core Facility of Memorial Sloan Kettering Cancer Center and The Rockefeller University as previously described [17]. All procedures were approved by the institutional animal care and use committee (protocol 97-03-009) and were carried out in accordance with National Institutes of Health (NIH) standards.

Selection of a conformational mAb with reactivity to oligomers presenting with a dominant β-sheet secondary structure was done by enzyme-linked immunosorbent assay (ELISA) analysis as previously described [17–19]. Plates were coated overnight at 4 °C with either Aβ_1–40_ or Aβ_1–42_ in 50 mM ammonium bicarbonate, pH 9.6, maximizing the oligomeric content difference between both Aβ_40_/Aβ_42_ peptides (Additional file 1: Figure S1), paired helical filaments (PHFs) purified from a human AD brain, or protease-resistant prion protein (PrP^Res^) as previously described [17, 19]. Approximately 125 μl of cell supernatant was diluted 1:1 with 50 mM Tris-buffered saline, pH 7.2, with 0.1% Tween 20 (TBS-T), and 50 μl/well were applied to each one of the four Immulon 2 HB (Thermo Fisher Scientific, Waltham, MA, USA) 96-well microtiter precoated plates. Bound antibodies were detected in the original cloning with horseradish peroxidase (HRP)-labeled goat antimouse immunoglobulin G (IgG) + IgM + IgA (H + L) (SeraCare Life Sciences, Gaithersburg, MD, USA). The color-developing substrate used was 3,3′,5,5′-tetramethylbenzidine (TMB; Pierce Biotechnology, Rockford, IL, USA), and readings were taken at 450 nm. aβComAb GW-23B7 was further subcloned, and levels of reactivity were tested as above in duplicates diluted 1:1000 in TBS-T and detected with HRP goat antimouse IgM(μ) (SeraCare Life Sciences) or HRP goat antimouse kappa chain (SouthernBiotech, Birmingham, AL, USA). Control coating was assessed with commercial 4G8/6E10 antibodies specific for Aβ peptides [20], PHF-1 for PHFs [21], and 7D9/6D11 antibodies for PrP [22, 23], all detected with HRP goat antimouse IgG (H + L) (GE Healthcare, Little Chalfont, UK). Duplicates of the controls were used to determine the lack of cross-reactivity of the secondary anti-μ with the IgGs or the coating proteins/peptides.

### Purification of aβComAb GW-23B7

The aβComAb GW-23B7 present in the subcloned hybridomas was precipitated with saturated ammonium sulfate (SAS) 761.5 g/L at 21 °C. Samples were made 30% in SAS, incubated at room temperature (RT) for at least 4 h, and centrifuged at 14,000 × *g* for 15 minutes, and the precipitate was washed with a comparable volume of 30% SAS and stored at 4 °C until further use.

Partially purified aβComAb GW-23B7 IgM was further purified using CaptureSelect IgM affinity matrix (Thermo Fisher Scientific) containing a 14 kDa llama antibody fragment specifically recognizing human or mouse IgM but no other immunoglobulins (IgG, IgA) from any animal. The ligand coupled to *N*-hydroxysuccinimide (NHS)-activated Sepharose 4 Fast Flow (GE Healthcare Life Sciences, Pittsburgh, PA, USA) has a binding capacity of 2.5 mg of IgM per milliliter of matrix. Briefly, 1 ml of matrix in 20% ethanol was poured at RT into a 3-ml plastic column, equilibrated, and washed with at least 20 bed volumes of PBS, pH 7.2. The column was drained to the top before adding the sample diluted in PBS. The flow rate was established at 1 ml/minute, and the collected flow was passed again at least three times through the column to maximize binding. The column was then washed with 5 bed volumes of PBS before eluting the IgM with 1.5 bed volumes of 0.1 M glycine, pH 3.0, and the eluates were immediately neutralized with 1 N NaOH. A second 1.5 bed volume of 0.1 M glycine, pH 3.0, was added to release all the bound IgM, and the column was regenerated with 10 bed volumes of PBS to start the process again as needed. The eluted IgM was aliquoted and kept at −80 °C until use. Each batch of IgM aβComAb GW-23B7 was assessed for purity on blots with specific antisera and protein stain, as well as for antibody activity against oligomers run on gels or ELISAs as described above.

### Surface plasmon resonance

Measurements of the affinity of aβComAb GW-23B7 to Aβ oligomeric conformers and monomeric Aβ were determined by surface plasmon resonance (SPR) using previously described methods [24]. Experiments were performed using a Reichert SR7000DC refractometer system (Reichert Technologies, Depew, NY, USA) equipped with a CM5 sensor chip. Acetate buffer was used for immobilization of the peptide Aβ_42_ (ligand). We used Aβ_42_ for these binding studies (not Aβ_40_) because Aβ_42_ species are the major constituents of amyloid plaques, with Aβ_40_ species being more common in vascular amyloid [25, 26], although some reports suggest that Aβ_40_ species overall can be the most abundant in the AD cortex [27, 28]. Importantly, Aβ_42_ species are considered critical for seeding of amyloid deposits [4, 29]. Carboxymethyl dextran on a CM5 gold sensor chip was activated by injecting a solution of 10 mg/ml NHS and 40 mg/ml 1-ethyl-3-(3-dimethylaminopropyl)carbodiimide over the sensor chip surface for 7 minutes at a flow rate of 20 μl/minute. Then the ligand, 50 μg/ml hexafluoroisopropanol-treated synthetic peptide Aβ_42_ (monomeric Aβ), or glutaraldehyde-polymerized Aβ_1–42_ in 10 mM sodium acetate (NaAc), pH 4.5, was immobilized by injection over the surface for 20 minutes. The unreacted sites on the sensor chip surface were blocked by injection of 1 M ethanolamine, pH 8.5, for 10 minutes. Analytes containing different concentrations of aβComAb GW-23B7 in PBS with 0.05% Tween 20, pH 7.4, were injected over the surface, and the association and dissociation reactions were monitored. After each binding cycle, the surface was regenerated by a short, fast injection of 10 mM hydrochloric acid. The binding dissociation constant (K_D_) was determined using Scrubber software 2.0a (BioLogic Software, Campbell, Australia).

### Electron microscopy

Samples of Aβ_1–40_ and Aβ_1–42_ dissolved in ammonium bicarbonate, pH 9.6, as used for ELISA coating, or samples of fibrilized and polymerized Aβ_1–42_, PHFs, and protein kinase A-digested PHFs in 1 mg/ml in PBS, pH 7.4, were all applied, 3 μl of each, onto carbon-coated 400 mesh Cu/Rh grids (Ted Pella Inc., Redding, CA, USA) and stained with 1% uranyl acetate in distilled water (Polysciences, Inc., Warrington, PA, USA). Stained grids were examined under a Philips CM-12 electron microscope (Philips Healthcare, Andover, MA, USA) and photographed with a Gatan (4 K × 2.7 K) digital camera (Gatan, Pleasanton, CA, USA). On electron micrographs, the pixel size is ~ 3.3 Å/pixel, and the defocus is −0.7 μm or −768 nm.

### Infusion of 3 × Tg mice with aβComAb GW-23B7 or vehicle control

**Fig. 1:**
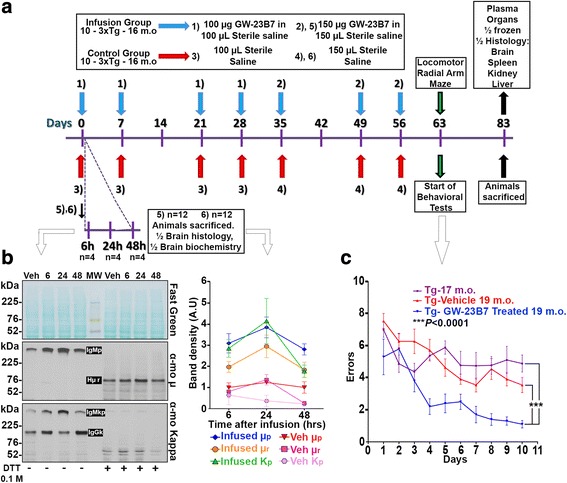
Protocol of infusion of GW-23B7 or vehicle control into triple-transgenic (3 × Tg) mice and the ensuing behavioral and kinetic tests. **a** Protocol of the intraperitoneal infusion of GW-23B7 or vehicle alone into 3 × Tg Alzheimer’s disease mice, behavioral tests, and killing of the tested animals. **b** Comparative kinetics of pentameric immunoglobulin M (IgM) distribution inside brains pooled from 18-month-old 3 × Tg animals infused with either GW-23B7 or vehicle alone as per the bottom part of protocol in (**a**). Western blots show soluble supernatants from 20% brain homogenates that were stained before and after reduction with reversible Fast Green to assess comparable protein loading (*top panel*) and detected by antimouse μ heavy chain or antimouse kappa light chain (*middle* and *bottom panels*, respectively). The relative concentrations determined by densitometry of the bands are plotted on the graph on the right. **c** Radial arm maze behavioral test showing significant differences (*p* < 0.0001 by two-way analysis of variance [ANOVA]) between Tg animals infused with GW-23B7 (*n* = 10) and both Tg vehicle-treated 19-month-old (*n* = 10) Tg mice at the baseline age of 16–17 months (*n* = 9). Dunnett’s multiple comparisons test showed no significant difference between the Tg mice at the baseline age of 16–17 months and the Tg vehicle-treated 19-month-old mice. There was also a significant time learning effect (*p* < 0.0001 by two-way ANOVA). *DTT* Dithiothreitol

The infusion protocol is illustrated in Fig. 1. All procedures were approved by the New York University (NYU) Institutional Animal Care and Use Committee (protocol 170202-01) and were carried out according to NIH standards. Two groups of ten of 16-month-old 3 × Tg AD mice (APP KM670/671NL [Swedish], MAPT P301L, PSEN1 M146V) [4] with amyloid and tau pathologies [30] received a weekly i.p. injection of either 100 μl of 1 μg/μl aβComAb GW-23B7 in sterile saline or 100 μl of sterile saline for the initial 2 weeks. One week later, the animals received the same weekly injections for the following 3 weeks. After 2 weeks, both groups were inoculated weekly with either 150 μl of 1 μg/μl GW-23B7 or 150 μl of sterile saline control for 2 more weeks. One week after the last injection, locomotor and behavioral cognitive tests were begun by one of the authors (AB), who was blinded to the experimental status of the mice. Mice were equally distributed throughout the behavioral testing, with mice from both experimental groups being represented in each day’s testing. Testing occurred over 3 weeks, after which the animals were bled from the heart, thoroughly perfused with PBS, pH 7.2, and killed. To avoid the possible stress of injections influencing the testing, the mice did not receive any infusions during the 3-week period of the locomotor and behavioral cognitive assessments. The brains were harvested immediately, the cerebellum was removed, and the remainder was split in half into two hemispheres, with one half flash-frozen over dry ice for future biochemical studies and the other half fixed in periodate-lysine-paraformaldehyde (PLP) for histochemistry. The intraperitoneal cavity was visually checked for anomalies, and the kidneys, spleen, and liver were collected for histological assessments.

Two additional groups of 18-month-old 3 × Tg mice (*n* = 12 each group) were used to assess the kinetics of IgM mAb penetration in the brain after a single peripheral injection. The animals in each group received an i.p. injection of either 150 μl of 1 μg/μl mAb GW-23B7 in sterile saline or 150 μl of sterile saline vehicle alone. Four animals of each group were killed at 6, 24, and 48 h and extensively perfused with PBS, then the brains were harvested for biochemical and histochemical assays as described above.

As part of the evaluation of different treatments in our 3 × Tg animals, a separate group of mice between the ages of 16 and 17 months (*n* = 9) were assessed cognitively using a radial arm maze. These 3 × Tg mice were from litters comparable to those of the mice that were used in the GW-23B7 infusion experiments.

### Locomotor and behavioral testing

Locomotor and behavioral cognitive tests were performed as previously described [18, 24]. Prior to each test, mice were allowed to adapt to the room lights for 15 minutes. Locomotor tasks were performed to verify that any treatment-related effects were not explained by sensorimotor abilities.

### Traverse beam

A traverse beam test was used to determine general motor coordination and balance. Each mouse was placed on a beam 1.1 cm wide and 50.8 cm long supported 30 cm above a padded surface, with a black box attached to the end of the beam. Mice were monitored for a maximum of 60 seconds, and the number of foot slips before falling or reaching the goal box was recorded. When an animal fell, it was placed back at the position where it was prior to the fall. The average number of foot slips per four successive trials was calculated. A foot slip was defined as an error and recorded numerically.

### Rotarod

Each animal was placed on a Rota-Rod 7650 accelerating model apparatus (Ugo Basile, Varese, Italy) with a diameter of 3.6 cm to assess differences in balance and forelimb and hind limb motor coordination. The animals were allowed to adapt to the apparatus for two training sessions and tested three times with increasing speed. The rotor rod was initially set at 1 rpm, and the speed was gradually increased every 30 seconds. The latency to fall or invert from the top of the rotating barrel was recorded. The rod was cleaned with water and 30% ethanol after each session.

### Radial arm maze

Spatial learning was evaluated using an eight-arm radial arm maze (arms 35 cm in length and 7 cm wide) with a cup of 1-cm diameter at the end of each arm. The central octagonal area had a 14-cm diameter and Plexiglas guillotine doors operated remotely by a pulley system. Each cup at the end of the arms was baited with saccharine-flavored water. The behavioral test consisted of 5 adaptation days followed by 10 trial days. All the animals subjected to the test were deprived of water (given access to water only 2 h/day). The trial time was set to a maximum of 15 minutes for each animal, and the time that the animals spent visiting all arms as well as the number of errors, defined as entries into previously visited arms, was recorded. All experimental groups were assessed concurrently, with the observer being blinded to the experimental status of the mice being tested.

### Brain homogenization

Flash-frozen brain hemispheres from each animal were weighted and made 20% wt/vol in tissue homogenization buffer (THB) containing 20 mM Tris, pH 7.4, 250 mM sucrose, 1 mM ethylenediaminetetraacetic acid, and 2.5 mM ethylene glycol-bis(β-aminoethyl ether)-*N*,*N*,*N′*,*N′*-tetraacetic acid filtered through a 0.2-μm mesh. Before use, freshly prepared 1.46 nM pepstatin, 1 mM phenylmethylsulfonyl fluoride, 1 mM sodium fluoride, and 0.96 mM sodium orthovanadate were added to obtain the working THB. Each half-brain in THB was placed on ice and homogenized using a PRO200 handheld homogenizer and a 5-mm × 75-mm flat-bottomed generator probe (PRO Scientific, Oxford, CT, USA) for three cycles of 30 seconds each at 30,000 rpm, with a 30-second pause between each homogenization cycle. The obtained 20% brain homogenates (BHs) were centrifuged twice at 25,000 × *g* for 10 minutes at 4 °C, and the supernatants were aliquoted (200 μl each) and stored at −80 °C.

Half-brains of two groups of 3 × Tg mice infused with aβComAb GW-23B7 or sterile saline and killed at 6, 24, and 48 h were homogenized as described above. Half of the samples of each group were pooled, aliquoted (200 μl each), and stored at −80 °C.

### Electrophoresis and Western blot analysis

#### Identity of monoclonal antibody

For electrophoresis to confirm the identity of aβComAb GW-23B7, 1 μg of antibody with or without dithiothreitol (DTT) 0.1 M was mixed with an equal volume of tricine sample buffer (Bio-Rad Laboratories, Hercules, CA, USA), electrophoresed on Bolt™ 4–12% Bis-Tris (Thermo Fisher Scientific) polyacrylamide gels and system, and transferred onto nitrocellulose membranes (NCs) for 1 h at 386 mA in 0.1% 3-(cyclohexylamino)-1-propanesulfonic acid (CAPS) (Sigma-Aldrich, St. Louis, MO, USA)-10% methanol. Equal protein loading was assessed by reversible protein stain Fast Green (FG) FCF 0.1% (Thermo Fisher Scientific) in 25% methanol-10% acetic acid for 1 minute, destained with 25% methanol, transferred to distilled water, and scanned on a Canon F916900 scanner (Canon Inc., Beijing, China). Membranes were then washed in TBS-T until the stain was eliminated, blocked 1 h at RT with 5% nonfat dry milk in TBS-T, pH 8.3, and incubated with HRP-conjugated rat antimouse IgM(μ) heavy chain-specific (1:6000; Thermo Fisher Scientific) or HRP-conjugated goat antimouse kappa (1:6000; SouthernBiotech). Bound antibodies were detected with an enhanced chemiluminescence (ECL) detection system (Pierce Biotechnology) on autoradiographic films (MIDSCI, St. Louis, MO, USA).

#### Reactivity of monoclonal antibody

To evaluate the reactivity of aβComAb GW-23B7 against Aβ_1–40_, Aβ_1–42_ (fibrillar and polymerized), and PHFs (fibrillar and protein kinase A-digested), 1–2 μg of each sample was electrophoresed, transferred to NC, and blotted as indicated above. Blots were incubated with aβComAb GW-23B7 diluted 1:1000 in TBS-T for 1 h at RT, and bound immunoglobulin was detected with HRP-conjugated antimouse IgM diluted 1:2000. Commercial anti-Aβ mAbs 4G8/6E10 (BioLegend, San Diego, CA, USA) and antiabnormally phosphorylated tau mAb PHF-1 (which recognizes phosphorylated serines at positions 396 and 404), kindly provided by Dr. Peter Davies (Feinstein Institute for Medical Research, Manhasset, NY, USA), were diluted 1:4000 and 1:2000, respectively, and used as controls for the identity of peptides Aβ_1–40_, Aβ_1–42_, and PHFs.

#### Measurement of IgM in brain soluble homogenates

To test for the presence of intact pentameric IgM kappa chain (IgMκ) in the brains of 3 × Tg mice treated with aβComAb GW-23B7 or with control sterile saline alone, pools of four 20% BHs corresponding to 6, 24, or 48 h postinjection were treated with SAS as follows: 165 μl of each pool were mixed with 135 μl of SAS, incubated for 30 minutes at RT in a tube rotator, and tubes were centrifuged for 6 minutes at 14,000 × *g*. Pellets were resuspended in 100 μl of 45% SAS, vortexed repeatedly, and centrifuged. Washed pellets were resuspended sequentially in 75 μl of distilled, deionized water and 75 μl of tricine sample buffer, then centrifuged for 5 minutes at 14,000 × *g* at RT. Quantities of 4 μl of the clear supernatant were mixed with 6 μl of tricine sample buffer with and without 0.1 M DTT and electrophoresed on Bolt™ 4–12% Bis-Tris polyacrylamide gels and system, and Western blot analysis was performed as previously described. Each sample represented the concentrated protein of ~ 1/150 of the whole brain to ensure that minimal representation or differences in IgM could be detected. aβComAb GW-23B7-infused animals were analyzed in pools and individually. The different bands were assessed densitometrically using ImageJ software (NIH, Bethesda, MD, USA). The same boxed area was used for each corresponding band.

#### Measurement of tau and Aβ oligomers in soluble brain homogenates

To quantitate the relative amounts of different oligomeric forms in the brains of control or treated animals, an aliquot of the 20% BH soluble supernatant corresponding to each one of the individual animals infused with aβComAb GW-23B7 or with control saline vehicle alone was thawed, and 2.5 μl were mixed with 2.5 μl of the tricine sample buffer, loaded onto an 8% Bolt^TM^ Bis-Tris polyacrylamide gel, electrophoresed on the Bolt^TM^ system, and transferred onto NC with CAPS buffer as described above. Afterward, the membranes were stained with FG reversible stain to assess comparable protein load, washed, blocked, and incubated sequentially first with PHF-1 antibody 1:1500 to detect p-tau oligomeric forms, followed by HRP antimouse IgG 1:3000 (GE Healthcare). Band detection was done with the ECL detection system (Pierce Biotechnology) on autoradiographic films (MIDSCI) with at least four different exposures for detecting the maximum number of bands in measurable densities. The positive and more relevant bands were scanned for recording and densitometric analysis as described above. The membrane was then stripped for 15 minutes at RT with gentle rotation in 12 ml of Restore^TM^ Western Blot Stripping Buffer (Pierce Biotechnology), washed three times with TBS-T, blocked as before for 10 minutes, washed three more times, and then incubated for Aβ oligomer detection with antibodies 4G8/6E10 1:4000 followed by HRP-conjugated antimouse IgG (1:4000; ECL system) at four different exposures on autoradiographic films, and scanned for documentation and densitometric analysis. As a final step, the membranes were stripped again, washed, blocked, washed, and incubated with the aβComAb GW-23B7 1:1000 in TBS-T, followed by incubation with HRP-conjugated antimouse IgM (1:2000; ECL system), with detection using autoradiographic films with four different exposures, followed by scanning for densitometric analysis. Densitometry was performed with ImageJ software.

### Quantitation of aggregated/oligomeric Aβ and phosphorylated tau in 3 × Tg mice

Aggregated/oligomeric Aβ levels were determined as previously described using the Human Aggregated Aβ ELISA Kit (Thermo Fisher Scientific) [18, 24] according to the manufacturer’s instructions. Briefly, 20% BHs were thawed and diluted 1:4 in the diluent buffer. Samples were applied to the ELISA plates and incubated for 2 h at RT, followed by extensive washing and incubation for 1 h at RT with biotin-conjugated detection antibodies that bind only to the immobilized aggregated Aβ. After removal of excess antibody, HRP-labeled streptavidin was added. Samples were incubated for 30 minutes, washed, and incubated with TMB substrate for color. The intensity of the colored product is directly proportional to the concentration of aggregated/oligomeric Aβ in the sample. The standards produced a linear curve, and the best-fit lines determined by linear regression were used to calculate aggregated Aβ concentrations in the samples.

For the quantification of total tau and phosphorylated tau (Thr231), the Meso Scale Discovery (MSD) assay (MSD, Rockville, MD, USA) was used as previously described [18]. Soluble supernatants of 20% BH from 3 × Tg treated and control mice were diluted 1:125 with the provided standard diluent buffer, and 100-μl aliquots were seeded into each well of a MULTI-SPOT® 96-well 4-spot plate (MSD). The plates were incubated for 2 h at RT, washed four times for 25 seconds each, and incubated for 1 h with the SULFO-TAG™ Anti-Total Tau antibody (MSD). The plates were then washed four times, covered to block the light, and incubated for 25 minutes with HRP-streptavidin working solution. The reaction was stopped with stop solution, and the plates were read on the MSD system at 450 nm. All data were recorded and calculations made using the software provided with the MSD system.

### Immunohistochemistry

Immunohistochemistry to assess the reactivity of aβComAb GW-23B7 was performed on old 3 × Tg mice (>19 months old) with extensive Aβ and tau pathology or on formalin-fixed, paraffin-embedded human cortex sections of AD, age-matched controls, young control brains, and Gerstmann-Sträussler-Scheinker syndrome (GSS) brains with prion pathology obtained from the Alzheimer brain bank of the Alzheimer’s Disease Center at NYU. All human tissue-related studies were done according to appropriate ethical standards under a protocol approved by the institutional review board at NYU School of Medicine. Written informed consent for research was obtained from the patients or their legal guardians. All data and samples were coded and handled according to NIH guidelines to protect patients’ identities. Sections were dewaxed, followed by rehydration with successive washes of xylene (two times, 5 minutes each), 100% ethanol (two times, 5 minutes each), 95% ethanol (5 minutes), 70% ethanol (5 minutes), and PBS (5 minutes). Next, slides were washed with 0.3% hydrogen peroxide (two times, 15 minutes each) to quench endogenous peroxidases and with PBS (three times, 5 minutes each), followed by 1-h blocking at RT with 10% normal goat serum (NGS) (Thermo Fisher Scientific)-0.2% Triton X-100 (Sigma-Aldrich) in PBS. Slides were then incubated overnight at 4 °C with aβComAb GW-23B7 diluted 1:2000 in 3% NGS-0.2% Triton X-100. Slides were washed three times with PBS and incubated with biotinylated antimouse IgM (μ-specific chain) antibody (Vector Laboratories, Burlingame, CA, USA) diluted 1:1000 in PBS for 1 h, followed by 1-h incubation in VECTASTAIN® AB solution (Vector Laboratories). Slides were developed with 3,3′-diaminobenzidine tetrahydrochloride with 2.5% nickel ammonium sulfate (Acros Organics, Morris Plains, NJ, USA) diluted in 0.2 M NaAc, pH 6. The reaction was stopped by removal of nickel solution and extensively rinsing with 0.2 M NaAc, then stabilized with PBS and mounted onto glass slides with Depex® Mounting Medium (Electron Microscopy Sciences, Hatfield, PA, USA).

For immunofluorescence, paraffin-embedded human AD or GSS brain slides were dewaxed and rehydrated as described above, followed by citrate buffer boiling for antigen retrieval, then blocked for 1 h at RT with 3% NGS-0.2% Triton X-100. Antibodies to be used for colocalization were diluted in the same tube containing PBS at the following concentrations: aβComAb GW-23B7 1:8000, anti-Aβ antibodies 4G8/6E10 1:3000, PHF-1 1:1500, and anti-glial fibrillary acidic protein (anti-GFAP) 1:1500. Primary antibodies were incubated overnight at 4 °C. Slides were washed with PBS (three times, 5 minutes each) and incubated for 2 h with Alexa Fluor® 488 (Thermo Fisher Scientific)-conjugated goat antimouse IgM (for GW-23B7) and Alexa Fluor® 647 (Thermo Fisher Scientific)-conjugated goat antimouse IgG (for commercial antibodies against proteins present in NDDs) (Jackson ImmunoResearch, West Grove, PA, USA), both diluted 1:500 in PBS. Slides were washed (three times, 5 minutes each) and coverslipped with PermaFluor™ Aqueous Mounting Medium (Thermo Fisher Scientific).

Black and red staining was performed using 3,3′-diaminobenzidine (DAB) for aβComAb GW-23B7 1:8500 as mentioned above. Slides were then washed with PBS (three times, 15 minutes each), blocked for 10 minutes at RT with 3% NGS-0.2%, incubated overnight with anti-Aβ antibodies 4G8/6E10 diluted 1:3000, washed with PBS (three times, 5 minutes each), and incubated with alkaline phosphatase antimouse IgG (Sigma-Aldrich) for 1 h, and color was developed with Vector Red Substrate (Vector Laboratories). Slides were washed with PBS and mounted onto glass slides as described above.

All mouse brain sections were fixed with PLP (40 μm each) and stained as previously reported [22], then incubated overnight with mAbs 4G8/6E10 1:3000, PHF-1 1:1500, or aβComAb GW-23B7 1:3000. Secondary antibodies used were biotinylated antimouse IgG (H + L) or antimouse IgM (Vector Laboratories) diluted 1:1000 in PBS. Slides were developed with DAB in 2.5% nickel ammonium sulfate and mounted onto glass slides with Depex® Mounting Medium.

### Statistical analysis

Statistical analyses were performed using Prism version 7 software (GraphPad Software, La Jolla, CA, USA). For the behavioral testing, two-way analysis of variance (ANOVA) was used, and two-tailed, unpaired *t* tests were used for biochemical assays.

## Results

The original clone 23B generated after the inoculation of a CD-1 mouse with β-sheet dominant p13Bri antigen and subsequent hybridoma production was assessed using an ELISA designed to detect the differential concentration of Aβ oligomers found in Aβ_1–40_ and Aβ_1–42_ samples aged in alkaline buffer and a PHF preparation rich in oligomers extracted from the brain of a confirmed typical human AD case (Additional file 1: Figure S1a and b). These misfolded proteins have no primary sequence homology and only share a common β-sheet secondary structure of the abnormal conformers.

The availability of only a small volume of cell supernatant from the initially viable hybridoma cells allowed only one determination per sample per each antigen and detection of all the main classes of immunoglobulins together by total goat antimouse immunoglobulins antisera. The selection process was previously described and involved the measurement of the differential reactivity to Aβ_40_, Aβ_42_, and PHF measured at least three times over the background optical density readings compared with an irrelevant hybridoma clone from the same fusion (Additional file 1: Figure S1b) [17]. The 23B clone passed three rounds of selection before being classified as GW-23B7 aβComAb. The GW-23B7 supernatant was found to contain two main proteins consistent with the albumin from the bovine fetal serum supplement (bovine serum albumin [BSA]) and an immunoglobulin with a high molecular weight of ~ 1000 kDa (Additional file 1: Figure S1c). The BSA was removed by partial purification on SAS precipitation, and Western blot analysis of the purified fraction showed a high-molecular-weight band on top of the gel before reduction and three bands of approximately 76 kDa, 56 kDa, and 27 kDa after reduction with DTT (Additional file 1: Figure S1d). The high-molecular-weight band reacted with both specific antimouse μ chain and antimouse kappa chain, as expected for an intact pentameric IgMκ. A small percentage of a monomeric form at ~ 180 kDa was also seen. The reduced material showed the intact heavy chain μ (Hμ) at 76 kDa and a small fraction of truncated Hμ chain at 56 kDa, which is not uncommon in IgM preparations. The 27 kDa band was identified as the reduced kappa light chain (Additional file 1: Figure S1e).

**Fig. 2:**
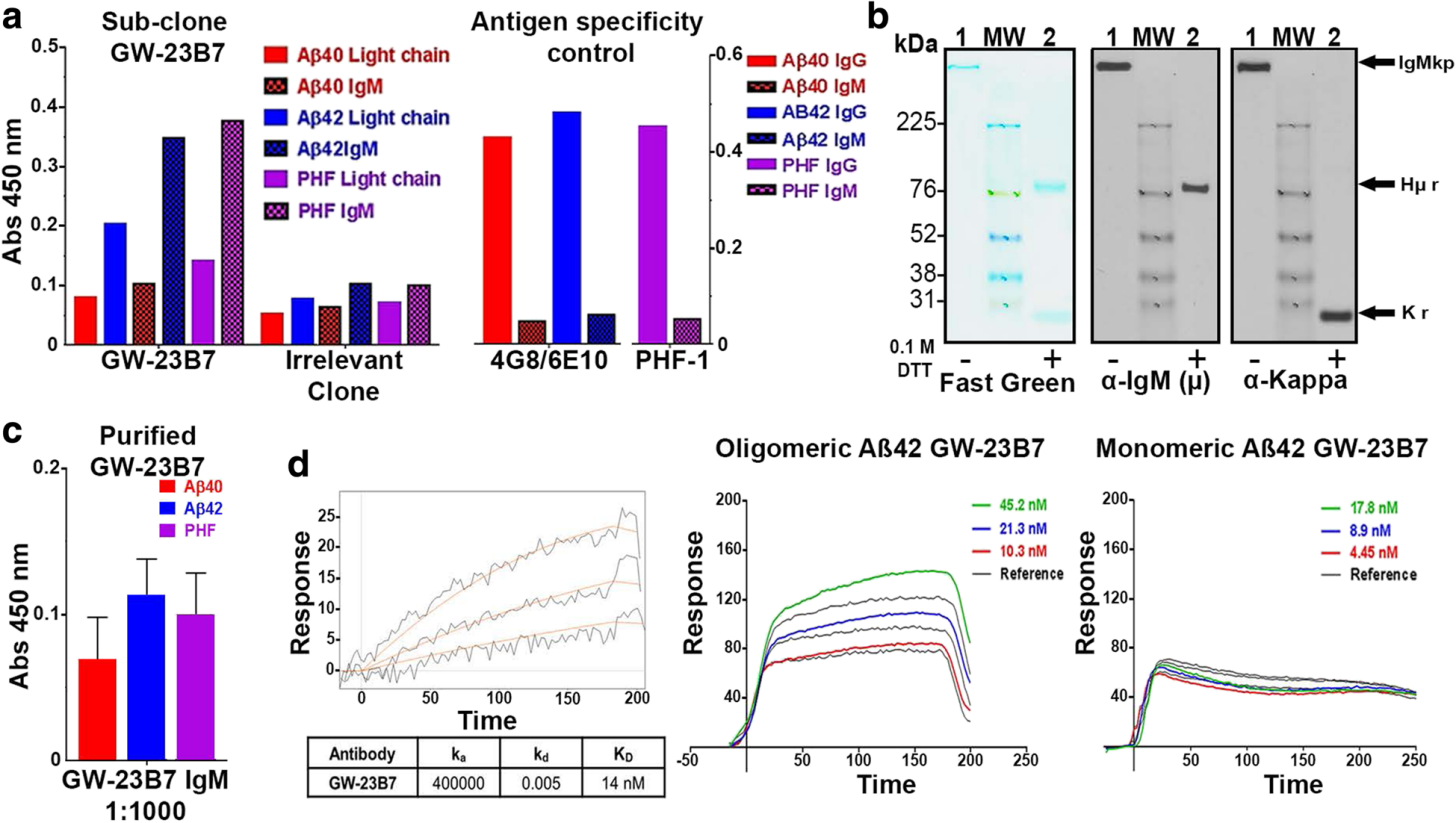
Subcloned and purified anti-β-sheet conformational monoclonal antibody (aβComAb) GW-23B7-specific reactivity to human paired helical filaments (PHFs) and oligomeric Aβ peptides. **a** Enzyme-linked immunosorbent assay (ELISA) data showing the reactivity from cell supernatant of subcloned GW-23B7 immunoglobulin M (IgM) and an irrelevant clone from the same fusion to PHFs and oligomers differential on Aβ_1–40_ and Aβ_1–42_. The *right panel* shows the even coating of the selected peptides on the plate and the lack of unspecific reactivity to secondary antimouse IgM. **b** Western blots showing the pentameric integrity of the purified aβComAb GW-23B7. *Lane 1* = unreduced sample, *lane 2* = reduced with 0.1 M dithiothreitol (DTT). *Left panel*: Fast Green reversible protein stain. *Middle panel*: Antimouse IgM (μ-specific) light chain. *Right panel*: Antimouse kappa light chain. *IgMp* Pentameric immunoglobulin M, *Hμr* μ heavy chain reduced, *Kr* Kappa light chain reduced. **c** ELISA results showing the reactivity of purified aβComAb GW-23B7 diluted 1:1000 to Aβ_1–40_, Aβ_1–42_, and human PHFs. **d** Surface plasmon resonance showing the binding affinity of the purified aβComAb GW-23B7 to the oligomeric species of Aβ_1–42_ and the lack of binding affinity to the monomeric forms. The dissociation constant (K_D_) (14 nM) was determined from the raw data on the left

The subclone GW-23B7 showed significant cross-reactivity with both the differential Aβ oligomers and the oligomeric/fibrillar PHF (Fig. 2a, left panel). The control antibodies used to monitor the antigens coating the plates were monoclonal mouse IgG antibodies for Aβ, 4G8/6E10, that recognize only the primary sequence of Aβ_40_ and Aβ_42_ [20], as well as mouse IgG antibody PHF-1 specific for p-tau that reacts only to human PHF [21]. The bound IgG-specific antibodies were not detected by the secondary antibody specific for μ chain, thus excluding unspecific cross-recognition of the coating antigens by the labeled secondary antisera (Fig. 2a, right panel).

Further purification was done using a llama V_HH_ domain camelids among them llamas- have a special type of antibodies that only has to heavy chain variable domains without light chains and are referred as VHH  antimouse IgM column, producing GW-23B7 that was > 99% pure pentameric IgMκ with intact heavy and light chains (Fig. 2b). Additional confirmational ELISA studies were done which showed that this purified version of GW-23B7 retained the same cross-reactivity to differential Aβ oligomers and as the original supernatant after a 1:1000 dilution (Fig. 2c). SPR studies were done to confirm the specificity of GW-23B7 to oligomeric Aβ_42_. The GW-23B7 showed specific binding affinity only for the oligomeric form of Aβ with a K_D_ of 14 nM, with no significant binding to monomeric Aβ (Fig. 2d).

**Fig. 3:**
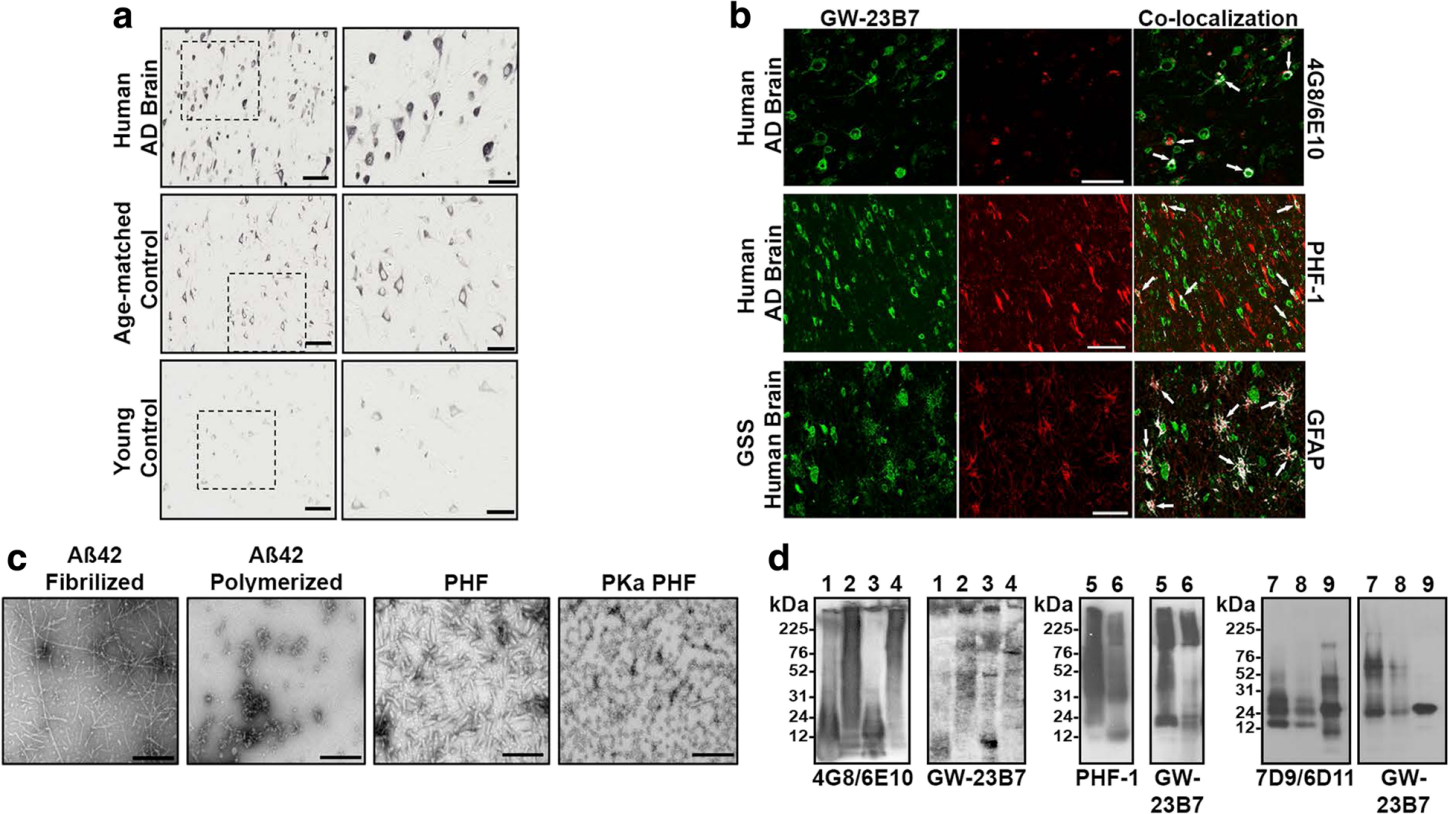
Histochemical reactivity of GW-23B7 to Alzheimer’s disease (AD), age-matched control, and young control human brains, and to Gerstmann-Sträussler-Scheinker syndrome (GSS) prion disease, as well as recognition of pathological conformers on immunoblots. **a** Representative immunohistochemical images showing reactivity of GW-23B7 on human AD brains (*top panels*) compared with age-matched and young human control brains (*middle* and *bottom panels*, respectively). *Right panels* are magnifications of the boxed areas on the left. Scale bars represent 100 μm on the left panels and 50 μm on the right panels. **b** Fluorescence immunohistochemistry of human AD brains (*two top panels*) and a GSS brain (*bottom panels*). *Left panels*: GW-23B7 (*green channel*). *Middle panels* (*red channels*): Commercial antibodies 4G8/6E10, PHF-1, and anti-glial fibrillary acidic protein (anti-GFAP), top to bottom, respectively. *Right panels*: Colocalization (indicated by *white arrows*). Scale bars represent 50 μm. **c** Electron microscopy of Aβ_1–42_ peptides fibrilized or polymerized with glutaraldehyde, paired helical filaments (PHFs), and protein kinase A-treated PHFs used on SDS-PAGE gels for immunoblots. **d** Immunoblots comparing reactivity of aβComAb GW-23B7 with the specific anti-Aβ peptide antibodies 4G8/6E10 to Aβ_1–40_, Aβ_1–40_ polymerized, Aβ_1–42_ fibrilized, and Aβ_1–42_ polymerized (*lanes 1–4*, respectively); the PHF-1 specific antibody for hyperphosphorylated tau on PHF and PHF protein kinase A-treated (*lanes 5 and 6*, respectively); and the 7D9/6D11 antibodies specific for prion protein (PrP) molecules to oligomerized PrP and chronic wasting disease prions (*lanes 7 and 8*, respectively)

Immunohistochemistry was used to determine which pathological features GW-23B7 was targeting. Brain sections from patients with AD, cognitively normal age-matched subjects, and young adults without neuropathology were stained. The GW-23B7 reacted strongly with intra- and extracellular material in the AD brains, consistent with dystrophic neurons and processes (Fig. 3a). The age-matched controls showed positive but much lighter cytoplasmic staining only in scattered neurons (Fig. 3a), and the brains from the young control subjects showed no immunoreactivity (Fig. 3a). The specificity of the GW-23B7 for human AD pathology was further analyzed using double-immunofluorescence staining to determine the nature of the cross-reactivity. GW-23B7 showed colocalization in the human AD brain with Aβ (visualized using 4G8/6E10 antibodies). Immunoreactivity was observed as scattered extracellular foci, as well as within a subset of neurons (Fig. 3b), most likely reflecting labeling of extra- and intracellular Aβ oligomer species. The latter is consistent with the reactivity of aβComAb GW-23B7 with the Aβ oligomer preparations characterized by electron microscopy (EM) and run on immunoblots (Fig. 3c and d, respectively). Colocalization of GW-23B7 was also observed with the anti-p-tau antibody PHF-1 in the cytoplasm of neurons (Fig. 3b). The pictures shown in Fig. 3 are representative of all sections analyzed from different parts of the brain with pathology. We examined sections from two age-matched and two young control subjects, as well as from three subjects with severe AD pathology (ABC [A for Aβ, B for Braak neurofibrillary tangle staging protocol, C for the Consortium to Establish a Registry for Alzheimer’s Disease neuritic plaque scoring system] score of A3B3C3 [31]). The cross-reactivity with p-tau oligomers was corroborated on immunoblots of EM-confirmed samples of oligomer-laced human PHFs (Fig. 3c and d, respectively). To further assess cross-reactivity with generic β-sheet in oligomeric conformers, samples from a brain of an unrelated NDD GSS, a human prion disease, were included. The GW-23B7 strongly colocalized with the typical histopathological gliosis of prion disease where the prion particles with high β-sheet content are associated with GFAP immunoreactivity in astrocytes (Fig. 3b, bottom panel). The identity of the reaction to PrP^Res^ was confirmed by immunoblot analysis (Fig. 3d).

The IgM aβComAb GW-23B7 was further analyzed in histological samples of human cortex and hippocampus of AD brains. The GW-23B7 detected, in both cortex and hippocampus, cytoplasmic labeling in neurons that retained cell shape and membrane integrity, neurons showing a dystrophic processes of varying severity, and neurons showing fragmentation with scattered punctuated material in the extracellular milieu. Labeling was also evident in some axons, dendrites, and synaptic boutons (Additional file 1: Figure S2a, b, c).

**Fig. 4:**
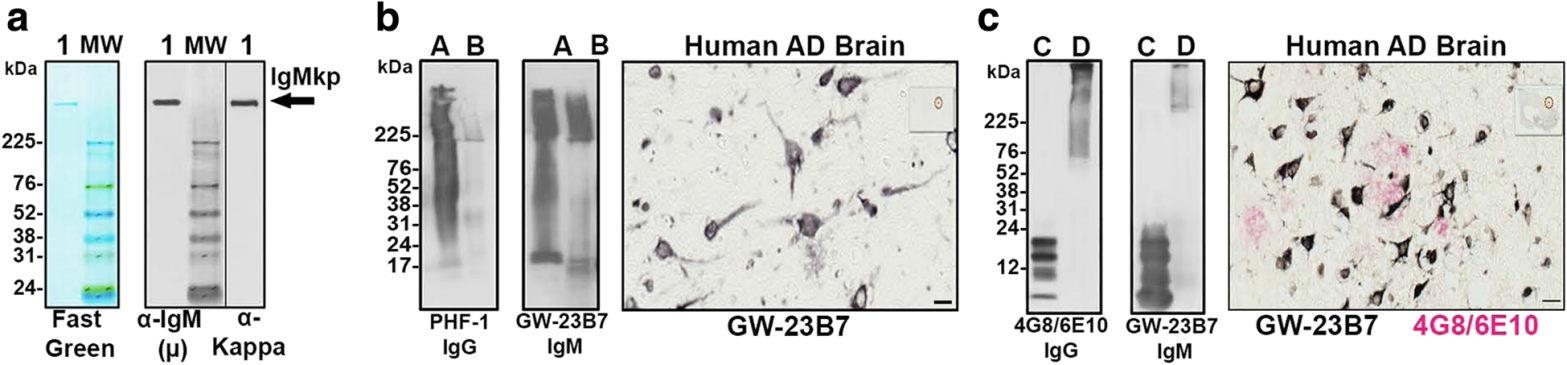
Molecular integrity and Alzheimer’s disease (AD) pathology: recognition of the CaptureSelect-purified GW-23B7 infused into old triple-transgenic mice. **a** Immunoblot showing protein stain with μ and kappa reactivity around the 1000 kDa position of an intact pentameric immunoglobulin M kappa chain (IgMκ) (*lane 1*). **b** Immunoblot of paired helical filaments (PHFs) and PHF protein kinase A purified from a human AD brain (*lanes A* and *B*) detected using a PHF-1 commercial antibody specific for hyperphosphorylated tau or using GW-23B7. *Right panel* shows immunohistochemical recognition by GW-23B7 of intra- and extracellular pathology-associated structures on a human AD brain section. **c** Immunoblots comparing reactivity to Aβ_1–42_ peptide and Aβ_1–42_ oligomerized (*lanes C* and *D*) by the commercial anti-Aβ 4G8/6E10 antibodies or GW-23B7; *right panel* shows colocalization on a human AD brain section of punctuated or strong precipitated material recognized by GW-23B7 (*black stain*) within amyloid plaques of Aβ detected by 4G8/6E10-specific antibodies (*red stain*)

To test the therapeutic potential of GW-23B7 to target pathological species in an AD mouse model, we selected the AD 3 × Tg mouse model with both Aβ and tau pathology [18, 30]. The GW-23B7 used in the in vivo experiments was documented to be > 99% pure intact IgMκ pentamer (Fig. 4a), to react with oligomeric/fibrillar forms of human extracted PHF in immunoblots and detect intra- and extracellular tau pathology in a human AD brain (Fig. 4b), to be cross-reactive with oligomeric forms of Aβ in immunoblots, and to colocalize with material related to amyloid plaques in a human AD brain (Fig. 4c). Our first aim was to ascertain whether pentameric IgM in this mouse model was able to cross the blood-brain barrier (BBB). Two groups of 12 animals each were infused i.p. as shown in the bottom part of the protocol in Fig. 1a. Four animals of each group were killed at 6, 24, and 48 h postinfusion. Each animal was thoroughly perfused with PBS to ensure all brain capillary vessels were devoid of residual immunoglobulin-carrying blood. A half-brain of each animal was homogenized and centrifuged to retain the soluble brain material (Fig. 1a).

To show that GW-23B7 was able to cross the BBB in an intact pentameric form, the soluble material was concentrated by at least 150 times, and the samples from each group were pooled for immunoblot analysis. Figure 1b shows a protein reversible stain (*top panel*) to ascertain whether there was a comparable protein load of each pool. The *middle panel* shows the antimouse μ heavy chain-specific reactivity before and after reduction: the intact pentameric IgM and the μ-chain at 76 kDa of the reduced IgMp. The *bottom panel* shows the reactivity with antimouse kappa light chain antisera detecting the intact IgMκ in the same pentameric position and also the normal polyclonal IgGκ at ~ 150 kDa. The densitometric analysis of each band is shown in the graph on the right of Fig. 1b. At all times checked, animals infused with GW-23B7 had a significantly higher concentration of pentameric IgM than the control group. The IgM in the GW-23B7-infused group was notable in the brain soluble material starting at 6 h postinfusion and peaking at 24 h, as demonstrated by both anti-μ and anti-kappa antisera, maintaining a substantial increase compared with controls after 48 h (Fig. 1b, *right graph*, and Additional file 1: Figure S4). The control group had similar concentrations of IgM at all time points, and all groups infused and controls had concentrations of IgG similar to the increased IgMκ in the GW-23B7-infused group, as determined by the kappa light chains detected in both classes of immunoglobulins (Fig. 1b, *bottom panel*, and Additional file 1: Figure S4), demonstrating that the IgM increase in the infused animals was not an artifact of the procedure. Our results document that pentameric IgM can cross the BBB and remain stable for more than 48 h at concentrations that are sufficient to have effects within the central nervous system, consistent with prior studies showing that a specific anti-Aβ IgM mAb can cross the BBB in association with acute benefits in learning impairment but not memory (at ~ 0.1% of the injected amount, which is similar to or better than IgG) and that IgM is a natural constituent of cerebrospinal fluid (CSF), suggesting that some systemic IgM normally crosses the BBB [32, 33]. Assuming that the infused antibody is allogeneic and catabolized in the same way as the rest of the mouse IgM, the peritoneal infusion permits a more regulated and smoother transition than the systemic route, preventing a transient overload in blood that could alter the half-life of the infused immunoglobulin. Each infusion was of 150 μg of intact pentameric IgMκ, and the same species was measured in the soluble portion of the brain to ascertain only the amount of the intact molecule that crossed the BBB (without measuring any degraded and likely ineffective antibody fragments, which could be included in other quantitative measures such as ELISA). Because the level of detection of a pentameric IgM on an immunoblot is approximately 5 ng, and because the analyzed samples represented a 150-fold concentration of the brain soluble material, the amount seen in excess of the normal intact pentameric IgMκ was on the order of 0.3% of the injected dose at 6 h postinfusion, > 0.5% at 24 h, and again ~ 0.3% at 48 h (Fig. 4b). These percentages are consistent with previously published results [32] for acute BBB penetration of an IgM.

Our second aim was to test the therapeutic efficacy of GW-23B7. To do so, injections were started in 16-month-old 3 × Tg mice, an age at which there is extensive established Aβ and tau pathology [18]. Two groups of ten animals each were infused i.p. for 2 months with either GW-23B7 in sterile saline or saline vehicle alone as per the protocol illustrated in Fig. 1a. At no time were any ill effects or sickness behaviors noted in the mice during the period of these injections. After 2 months, the 18-month-old mice were first subjected to locomotor testing. No differences between the GW-23B7-injected mice and vehicle-injected controls were noted in locomotor studies (Additional file 1: Figure S3). The two groups were subsequently tested in the radial arm maze; these two groups were also compared with a set of 16- to 17-month-old 3 × Tg animals (*n* = 9) from the same breeding pairs. The 19-month-old GW-23B7-treated animals showed a significant cognitive rescue compared with the vehicle control group, as well as with the 3 × Tg mice at the baseline age of 16–17 months (*p* < 0.0001 by two-way ANOVA) (Fig. 1c). Dunnett’s multiple comparisons test showed no significant difference between the 3 × Tg mice at the age of 16–17 months and the Tg vehicle-treated 19-month-old mice. Immediately afterward, all animals were killed at 19 months of age, and their organs and brains were collected and divided for histology and biochemistry.

**Fig. 5:**
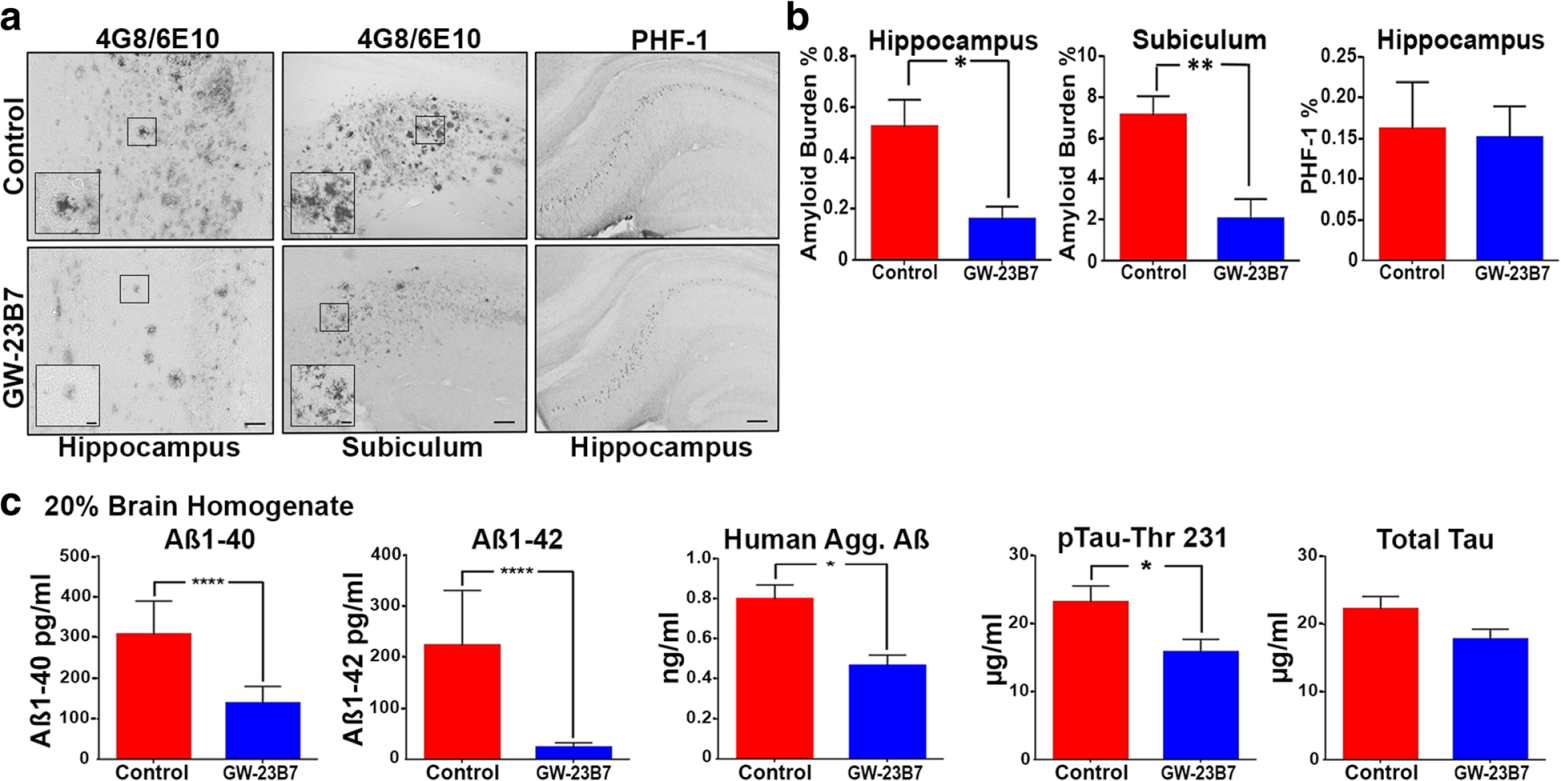
Levels of extracellular amyloid-β we are only measuring the amyloid burden and not the rest of A burden, intracellular paired helical filament (PHF) burden, and soluble Aβ or hyperphosphorylated tau (p-tau) in brains from 19-month-old triple-transgenic (3 × Tg) mice infused with GW-23B7 or control vehicle alone. **a** Immunohistochemistry of representative GW-23B7- or vehicle control-infused 3 × Tg mouse brains showing hippocampus and subiculum amyloid plaques as detected by specific antibodies 4G8/6E10 or intraneuronal PHF as detected by commercial antibody PHF-1. Scale bars for 4G8/6E10 hippocampus images represent 50 μm, and scale bars for 4G8/6E10 subiculum images represent 100 μm. Scale bars for insets are 10 μm. Scale bars for PHF-1 images represent 200 μm. **b** Quantitation of the amyloid and tau burden for each group of infused animals characterized in (**a**). *p < 0.05 and **p < 0.01. No significant differences in intracellular PHF-1 burden were found. **c** Levels of soluble Aβ_1–40_, Aβ_1–42_, human aggregated Aβ, threonine 231 phosphorylated tau, and total tau in supernatants from 20% brain homogenates of the GW-23B7- and vehicle-infused 3 × Tg mice groups. *p < 0.05 and **** *p* < 0.0001

Figure 5a shows representative samples of hippocampus and subiculum of brains from the GW-23B7-infused and control groups. The striking difference in the Aβ burden among GW-23B7-treated mice is shown Fig. 5b in the hippocampus and subiculum. However, quantitation of PHF-1 intracellular neuronal immunoreactivity did not show a significant difference between treated and control groups (Fig. 5a and b). The degree of tau-related pathology shown is representative of our cohort of 3 × Tg mice at these ages and is similar to what we previously reported [17, 18, 24, 34].

Biochemical assays of the BHs that would contain soluble oligomeric conformers showed that the total Aβ_40_ and Aβ_42_ levels were significantly reduced in the GW-23B7-infused animals, correlating with the reductions in the aggregated, deposited Aβ shown immunohistochemically (Fig. 5c), consistent with significant reductions in Aβ oligomers. The latter was confirmed by a notable reduction of Aβ oligomers documented by an ELISA specific for soluble aggregated Aβ species (Fig. 5c, *middle graph*). Importantly, a significant reduction in only abnormally phosphorylated soluble tau species was documented by measures of p-tau-Thr231 in the treated animals, whereas the total soluble tau showed no significant difference between the two groups of mice (Fig. 5c, *two right graphs*).

**Fig. 6:**
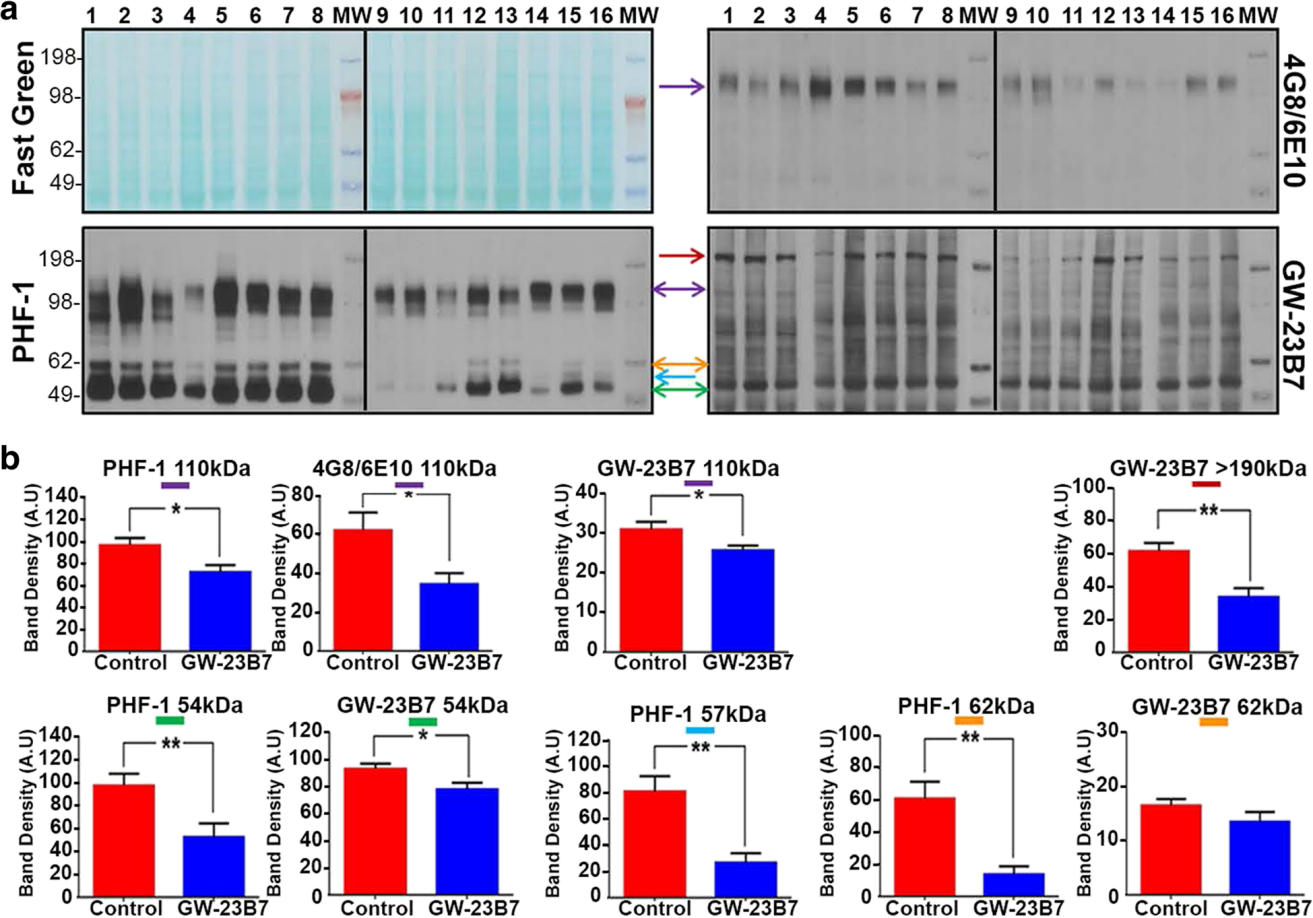
Immunoblots detecting oligomers on soluble supernatants from 20% brain homogenates of 19-month-old triple-transgenic (3 × Tg) mice infused with purified GW-23B7 or vehicle alone. **a** SDS-PAGE blotting of individual soluble supernatants from 20% brain homogenates of control infused 19-month-old 3 × Tg mice (*lanes 1–8*) and 19-month-old 3 × Tg mice infused with GW-23B7 (*lanes 9–16*). *Top left panel*: Fast Green protein reversible stain for comparable loading. *Top right panel*: Immunoblot with 4G8/6E10 antibodies specific for Aβ peptides. *Bottom left panel*: Immunoblot with PHF-1 antibody specific for hyperphosphorylated tau. *Bottom right panel*: Immunoblot with GW-23B7. Oligomeric forms analyzed were of different molecular weights and are identified by *color-coded arrows*; same color = same molecular weight. **b** Densitometric quantitation of the oligomer bands coded with *colored arrows* in (**a**). Statistical analysis was done using two-tailed, unpaired *t* tests. * *p* < 0.05; ** *p* < 0.01

To assess the possible identity of the soluble oligomeric forms of Aβ and tau that were modified by immunotherapy, the soluble portion of the BHs was run on gels and blotted (Fig. 6a). The membranes from representative animals from both groups and stained for total protein with a reversible FG showed comparable sample loads among all samples in an extended range of molecular weights (Fig. 6a, *top left panel*). This membrane was blotted with 4G8/6E10 to detect aggregated Aβ (Fig. 6a, *top right panel*), with PHF-1 used to detect abnormal tau conformers (Fig. 6a, *bottom left panel*) and with the same GW-23B7 that was used for immunotherapeutically infused animals (Fig. 6a, *bottom right panel*). The potential oligomeric forms analyzed were color-coded and quantitated densitometrically after scanning of films. The graphed results are shown in Fig. 6b. Only one band (purple) at 110 kDa showed colabeling for both Aβ and tau and was also recognized by GW-23B7. All three antibodies showed a significant decrease of this hetero-oligomer in the treated animals (*top row*, three left purple-coded graphs). From 54 to 62 kDa, three bands were detected by PHF-1 and showed a significant decrease in the GW-23B7-treated animals (*bottom row graphs*). The 54 kDa (*green*) decrease was also confirmed by GW-23B7, the 57 kDa (*blue*) could be measured only with PHF-1, and the decrease of the 62 kDa (*orange*) was significant only when measured by PHF-1. A band of high molecular weight of ~ 190 kDa (*red*) was seen only with GW-23B7 and was significantly decreased in the treated animals.

## Discussion

Understanding of the pathogenesis of various NDDs, and AD in particular, has increased exponentially in recent years; however, despite this growing knowledge base, translating preclinical therapeutic successes with animal models to the clinic remains an elusive goal [16, 35]. Immunotherapy was deemed to be a major potential option; however, thus far, trials have shown poor or discouraging results, with the failure rate of AD-related clinical trials being ~ 99.6% [16, 36–40]. This high failure rate may be related in part to the targeting of either Aβ or tau pathology and not both pathologies, as well as to the lack of specific targeting of oligomeric species. Extensive evidence indicates that oligomers of both Aβ and tau are the most neurotoxic species in AD, because levels of these species correlate better with cognitive decline than the burden of either plaques or NFTs [4, 41, 42]. Our approach for the potential treatment of AD follows the rationale that only a therapy addressing both these toxic species and their related pathologies may have some chance of success [17].

The first principle underlying this rationale is to have an immune reaction to a generic trait present in both Aβ and tau pathology conformers. These two proteins have no sequence homology and share in common only a dominant β-sheet secondary structure produced during the conversion of the self physiological proteins/peptides to the pathologic oligomeric conformers, a conformation that is also present in PrP^Res^ and α-synuclein oligomers. To accomplish this goal, we developed an immunogen with no sequence similarity to any mammal protein/peptide and polymerized it into oligomers with > 90% β-sheet structure and no tendency to develop fibrillary forms [17–19]. This immunogen was proved to prevent pathology in three different mouse models of AD covering the whole range of Aβ, tau, and vascular amyloid pathologies [11, 18, 19]. It was used to inoculate mice and produce hybridomas selected exclusively with at least three different neuroconformers having in common only β-sheet-dominant structures in their oligomers [17]. We isolated a few families of mAbs that cross-reacted with different pathological conformers and called them *secondary structure aβComAb* to differentiate them from other anticonformational mAbs raised against a specific primary and/or tertiary structure that produces more limited cross-reactivity [17].

In this report, we focus on aβComAb GW-23B7, which we have extensively studied and purified, and it showed a good binding affinity to oligomeric forms of Aβ_1–42_, with no binding to the same Aβ_42_ peptide in a monomeric form, as well as binding to oligomers from PHF extracted from a human AD brain (Figs. 2 and 3 and Additional file 1: Figures S1 and S2). The potential anti-β-sheet cross-reactivity was confirmed by also detecting prion oligomers and showing colocalization with different pathological components in human brains with AD and prion disease (Fig. 3). To test the therapeutic potential of GW-23B7 in vivo, GW-23B7 was first purified and shown to still conserve its pentameric integrity and specific cross-reactivity profile (Fig. 4).

Importantly, we show that the intact pentameric GW-23B7 is able to cross the BBB with a peak at 24 h after i.p. injection and remain at stable elevated levels with immunoglobulin integrity after 48 h (Fig. 1 and Additional file 1: Figure S4). Our findings are consistent with a prior study showing that a specific anti-Aβ IgM mAb, L11.3, can cross the BBB; however, the latency of our GW-23B7 in the brain of the mice was significantly longer, likely related to the fact that L11.3 is a xenogeneic IgM mAb tested in a mouse model [32]. In this study, ~ 0.1% of the injected IgM mAb crossed the BBB, which exceeded the BBB passage of an anti-Aβ IgG mAb [32]. Interestingly, the L11.3 produced an acute improvement in learning skills but did not improve memory; behavioral benefits were much more limited than with GW-23B7. Nevertheless, this past study also demonstrated that an IgM can cross the BBB at concentrations sufficient to have some therapeutic effects. It is also well established that IgM is a natural constituent of CSF, suggesting that some systemic IgM normally crosses the BBB [33, 43]. Hence, although the vast majority of therapeutic antibodies are IgGs, our findings suggest that it would be possible to use an mAb IgM for AD therapy. Moreover, specific carriers have been reported to greatly enhance the BBB passage of intact IgM, potentially enhancing therapeutic efficacy [44]. Our relatively short treatment period of GW-23B7 injections resulted in significant cognitive benefits in 3 × Tg mice with advanced Aβ and tau pathologies. This cognitive amelioration was not confounded by any locomotor differences between treated and control groups, because we established that the two groups of mice performed similarly on rotarod and traverse beam testing prior to the radial maze cognitive assay (Fig. 1 and Additional file 1: Figure S3).

This behavioral rescue in treated mice correlated with reductions in the amyloid burden quantitated by immunohistochemistry as well as biochemical reductions of total Aβ_40_/Aβ_42_ in the soluble BH fraction (Fig. 5). We also documented reductions of aggregated, soluble Aβ species by ELISA. Importantly, GW-23B7 treatment did not reduce intracellular NFT neuronal pathology, although it reduced pathology-associated extracellular hyperphosphorylated and oligomeric tau species (Figs. 5 and 6). The IgM GW-23B7 likely has limited access to the intraneuronal compartment; hence, there is limited reduction of the relatively inert deposited tau hyperphosphorylated species in the form of NFTs. Numerous studies suggest that tau pathology spreads through a “prion-like” mechanism via extracellular tau oligomeric species [1–7]. Hence, we document that GW-23B7 treatment reduces the levels of the most relevant pathology-associated species of both Aβ and p-tau with minimal risk for associated neuronal toxicity.

Interestingly, we document the presence of an oligomeric species at ~ 110 kDa that appears to be a hetero-oligomeric species of Aβ and tau that is reduced by GW-23B7 treatment (Fig. 6). Although Aβ accumulation is generally viewed as upstream from tau pathology, there is extensive evidence of a significant interplay of these two pathologies. Numerous pathogenetic aspects of transgenic AD models can be rescued by ablating the tau gene [45–47]; however, other studies show that aggregated Aβ can enhance tau pathology or conversely that aggregated tau may enhance Aβ toxicity [47–50]. A potential advantage of mAbs such as GW-23B7 that target both Aβ and tau oligomeric species is that they may be particularly effective at inhibiting such detrimental positive feedback loops. Moreover, the aβComAb GW-23B7 detected a high-molecular-weight oligomer at ~ 200 kDa that was not detected by either commercial antisera to Aβ or p-tau (Fig. 6). The epitopes detected by these standard antibodies were likely buried within this large oligomeric species, whereas the pathology-associated β-sheet secondary structure was still being detected by GW-23B7. This oligomeric form was significantly reduced in the treated animals, suggesting its possible importance for mediating cognitive benefits. Regardless of the potential toxicity of this oligomeric species, the ability of GW-23B7 and other related aβComAb to detect previously unseen high-molecular-weight oligomers in different NDDs opens the door to new studies to better define different pathology-associated oligomers [17]. An additional potential advantage is that mAbs that recognize the shared β-sheet secondary structure found in many different oligomers may concurrently target Aβ-, tau-, and α-synuclein-related pathologic conformers, addressing the mixed pathologies found in the majority of patients with NDD [51–54].

## Conclusions

The pathogenesis of AD is complex, with genome-wide association studies and other genetic studies showing that dysfunction of various pathways can lead downstream to the accumulation of both toxic Aβ and tau oligomeric species [35, 55]. Thus far, various therapeutic approaches that address either Aβ or tau pathology separately have proved disappointing in trials [12, 40]. We show that aβComAb can reduce both Aβ and tau oligomeric species in association with cognitive benefits. Such combined multiple-target approaches may have a greater chance of future clinical therapeutic success.

## Additional file

Additional file 1: Figure S1. Reactivity of the original hybridoma 23B selected clone against Aβ_1–40_, Aβ_1–42_, and PHF and the partial purification of the subclone GW-23B7 with saturated ammonium sulfate (SAS). **Figure S2.** Conformational monoclonal antibody GW-23B7 immunoreactivity in human Alzheimer’s disease brains. **Figure S3.** Locomotor tests on 18-month-old 3 × Tg AD mice infused with aβComAb GW-23B7 or with control vehicle alone. **Figure S4.** Individual IgMκ measurements in brain homogenate supernatants of infused animals after 6, 24, and 48 h. (PDF 2160 kb)
