# Supplementary material for: Anti-β-sheet conformation monoclonal antibody reduces tau and Aβ oligomer pathology in an Alzheimer’s disease model
Source: Alzheimers Res Ther. 2018 Jan 29;10:10. doi: 10.1186/s13195-018-0337-3 (PMC5789573; doi:10.1186/s13195-018-0337-3)
Supplement: Additional file 1: Figure S1. — Reactivity of the original hybridoma 23B selected clone against Aβ1–40, Aβ1–42, and PHF and the partial purification of the subclone GW-23B7 with saturated ammonium sulfate (SAS). Figure S2. Conformational monoclonal antibody GW-23B7 immunoreactivity in human Alzheimer’s disease brains. Figure S3. Locomotor tests on 18-month-old 3 × Tg AD mice infused with aβComAb GW-23B7 or with control vehicle alone. Figure S4. Individual IgMκ measurements in brain homogenate supernatants of infused animals after 6, 24, and 48 h. (PDF 2160 kb) [file 13195_2018_337_MOESM1_ESM.pdf]

Figures S1-S4 for:

Anti-β-sheet Conformation Monoclonal Reduces Aβ and Tau Oligomer Pathology in an Alzheimer's Model

Fernando Goñi*[1], Mitchell Martá-Ariza[1], Krystal Herline[1], Daniel Peyser[1], Allal Boutajangout[1,3], Pankaj Mehta[4], Eleanor Drummond[1], Frances Prelli[1] and Thomas Wisniewski*[1,2,3]

[1]Center for Cognitive Neurology and Department of Neurology, New York University School of Medicine, New York, New York, USA. [2,3]Departments of Pathology and Psychiatry, New York University School of Medicine, New York, New York, USA. [4]Department of Immunology, New York State Institute for Basic Research in Developmental Disabilities, Staten Island, USA.

Correspondence should be addressed to T.W. (Thomas.Wisniewski@nyumc.org) or F.G. (Fernando.Goni@nyumc.org)

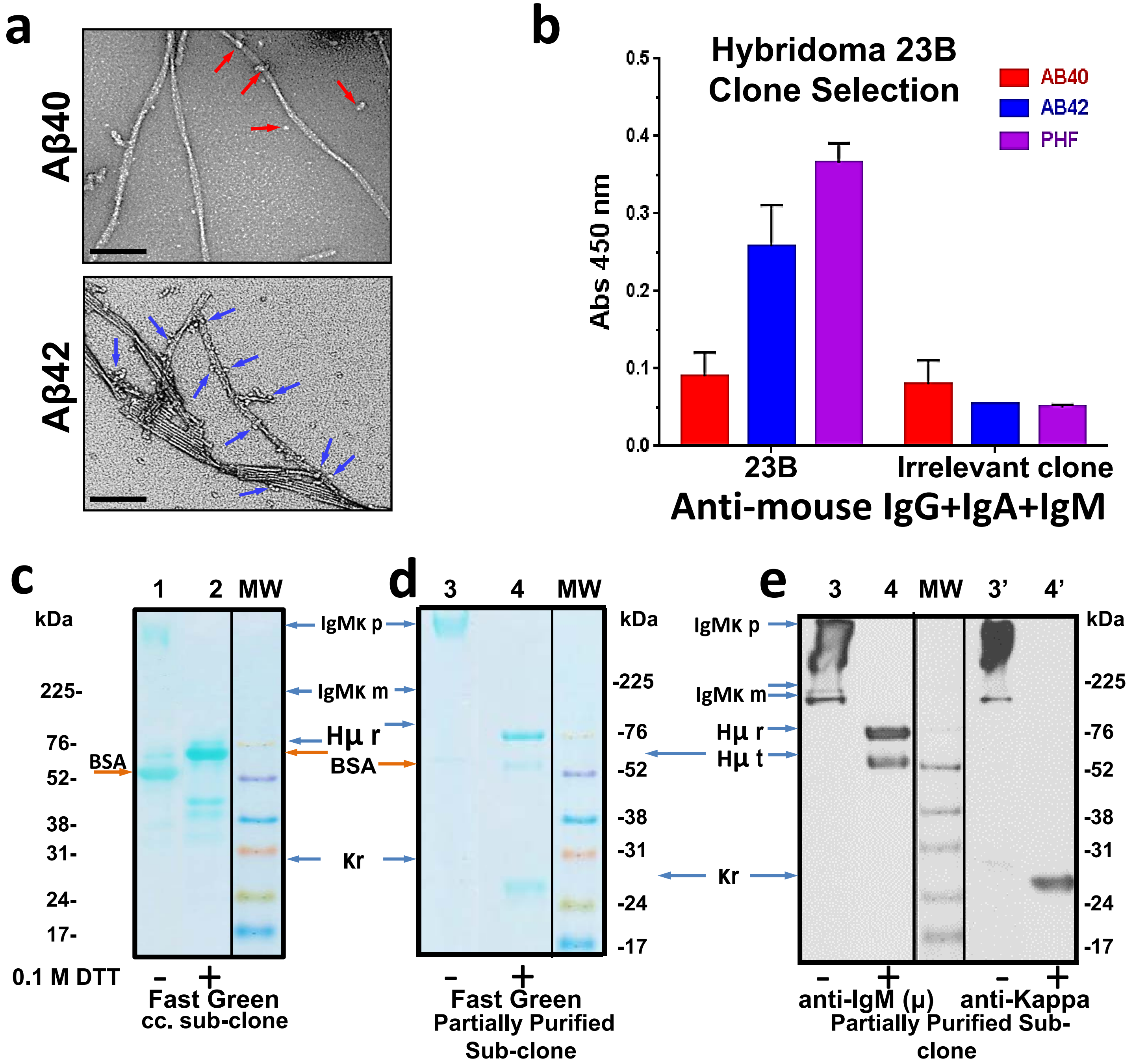

Figure S1. Reactivity of the original hybridoma 23B selected clone against Aβ1-40, Aβ1-42 and PHF; and the partial purification of the sub-clone GW-23B7 with saturated ammonium sulfate (SAS). a. Electron microscopy images of Aβ40 and Aβ42 . Red arrows show few oligomeric forms bound to the fibrils, whereas blue arrows depict extensive amount of oligomers associated to the amyloid fibrils. **b.** ELISA assay showing cross-reactivity of the cell supernatant of hybridoma 23B clone to Aβ1-40, Aβ1-42 and PHF detected by an anti-mouse IgG+IgA+IgM (H+L) antisera. **c.** Fast Green of the concentrated cell supernatant, unreduced and DTT reduced Lanes 1 and 2, obtained from the sub-cloned conformational mAb GW-23B7 before purification and dominated by Bovine serum albumin (BSA) from fetal calf serum. **d.** Fast Green of the 30% SAS precipitate showing the intact IgM and the Heavy and Light chains before and after reduction respectively (Lanes 3 and 4), and a small amount of the remaining BSA. IgMk p: pentameric; IgMk m: monomeric; Heavy chain reduced (Hμ r); truncated Heavy chain reduced (Hμ t), and Kappa Light chain reduced (Kr) shown. **e.** Immunoblot of the SAS partially purified conformational mAb GW-23B7. The anti-mouse IgM (μ chain specific) shows the intact pentamer and IgM monomer before reduction (Lane 3) and after reduction the Heavy chain intact around 76 kDa plus 10-15% of a truncated Heavy chain at 60 kDa (Lane 4). The anti-mouse kappa antibody shows its presence in the pentameric and slightly in the monomeric IgM before reduction (Lane 3') and only one band for the Kappa Light chain after reduction (Lane 4').

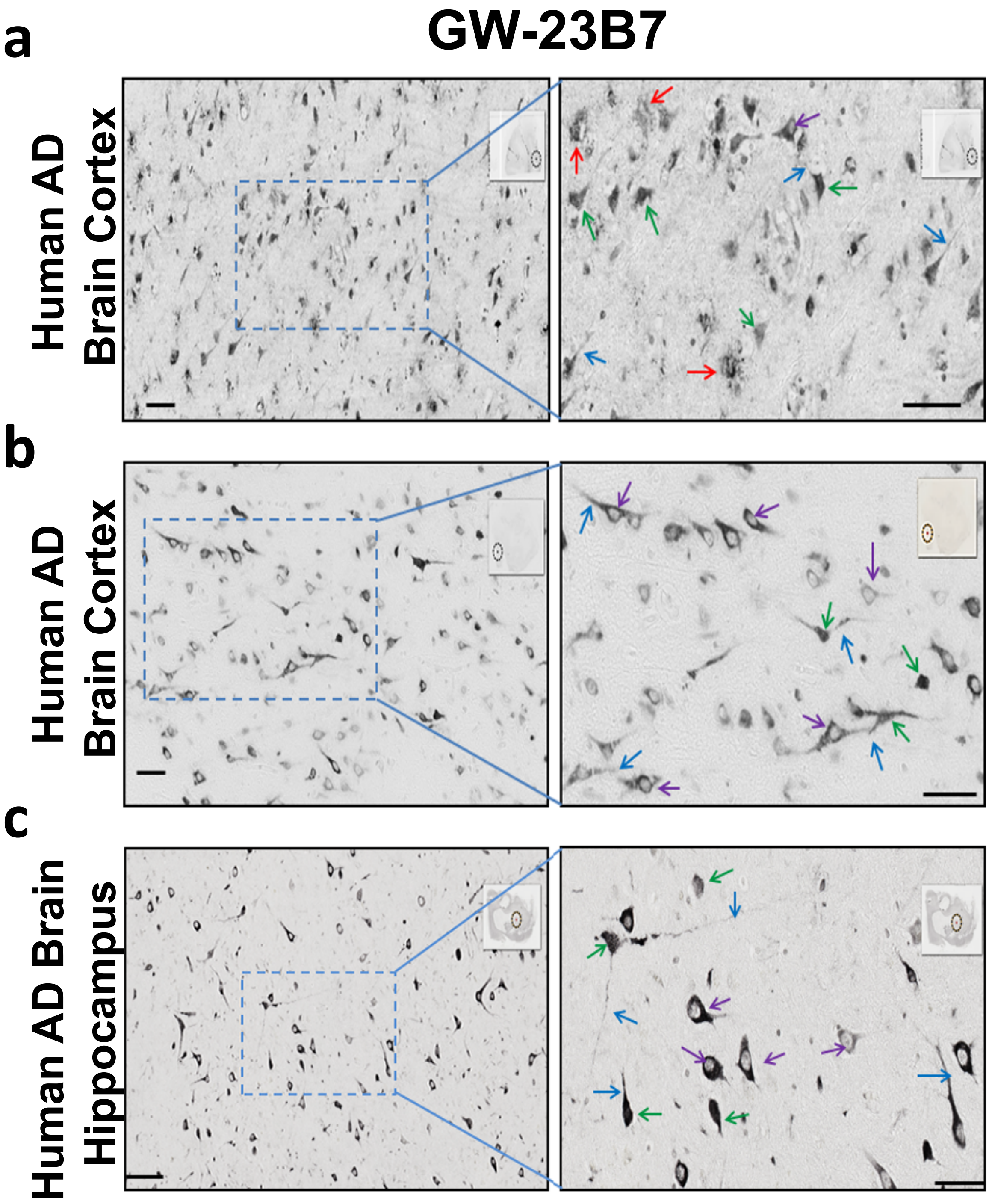

Figure S2. Conformational monoclonal antibody GW-23B7 immunoreactivity on human Alzheimer's disease brains. All slides were developed using DAB after conformational mAb GW-23B7 was used as primary reagent and HRP-coupled anti-mouse IgM (μ specific) as secondary. Right panels are magnifications of the boxed areas on the left. Bars represent 50 μm. a, b: Cortex; c: Hippocampus**. a, b, c.** Dark stain is seen in the cytoplasm of many neurons with some of them extending punctuate stain inside the nucleus (purple arrows). Panel **a)** shows extensive punctuated extra-cellular material (red arrows) coincidental where plaque contours are seen and more neurons seem to be dystrophic or degraded to a point that they lose membrane definition and integrity (green arrows). Neuronal processes are also detected (blue arrows) some, in panel **b)**, showing to make contact through zones of defined or undefined intact structure; whereas in panel **c)** the length of the detected material can travel in well-defined cellular structures as in the neuron on the bottom right (right panel) or with discontinuous structure "leaking" to the extracellular milieu from the dystrophic body of the neuron on the upper left.

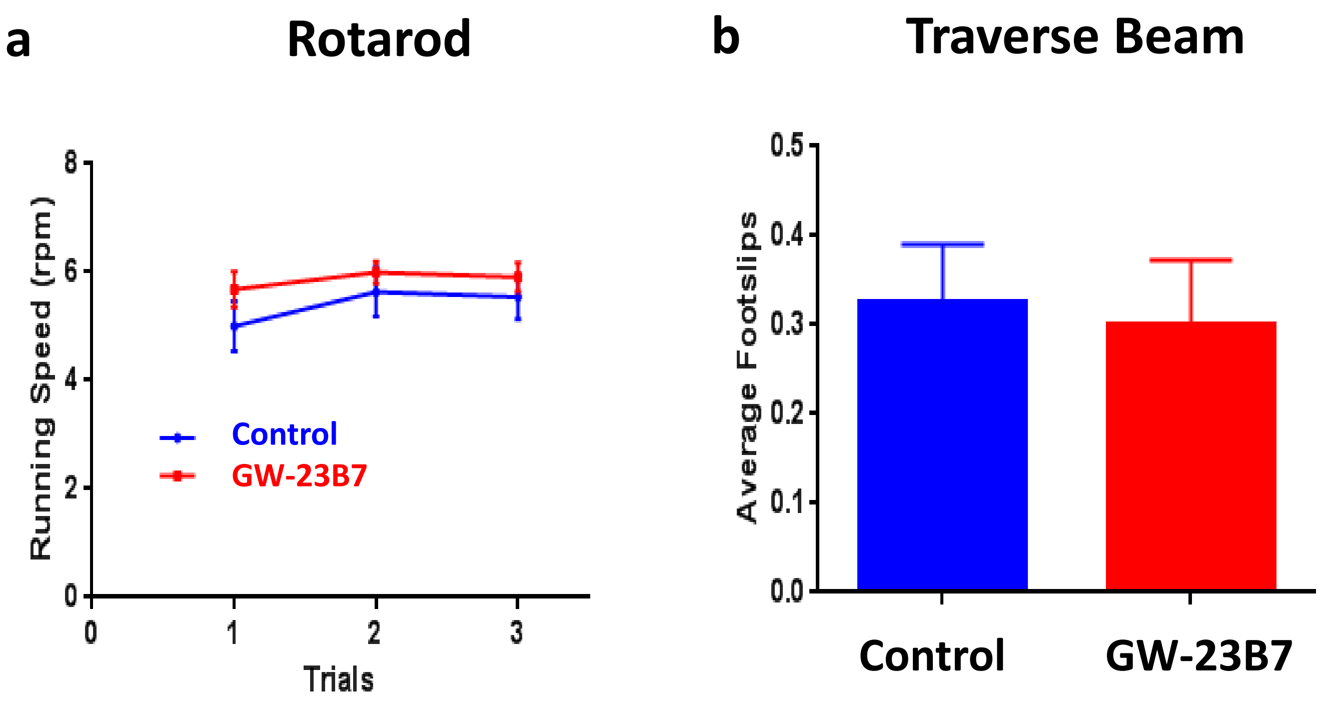

Figure S3. Locomotor tests on 18 m.o. 3xTg AD mice infused with aβComAb GW-23B7 or with control vehicle alone. a. Rotarod to determine balance and coordination; no significant differences were seen between the control and the GW-23B7 infused groups. **b.** Traverse Beam to determine general motor coordination; no significant differences were seen between the control and the GW-23B7 infused groups

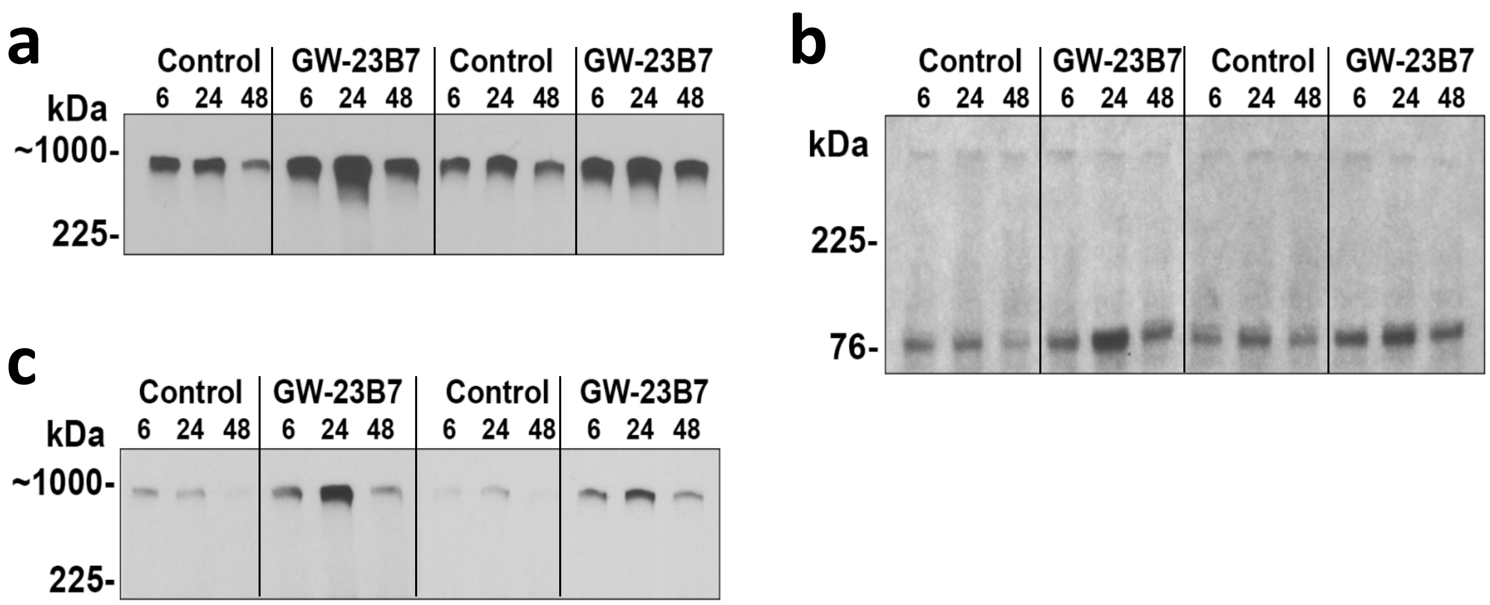

Figure S4. Individual IgMk measurements in brain homogenate supernatants of infused animals after 6, 24 and 48 hrs. a. Shows the pentameric IgM μ immunoblot reaction from two controls and two GW-23B7 infused animals sacrificed at 6, 24 and 48 hrs illustrating the marked differences in concentration between the two groups at each of the 3 time points, with a peak IgM concentration at 24 hrs in the GW-23B7 infused animals**. b**. Shows the same result after reduction with DTT and detection of the intact Hμ at a 76 kDa molecular weight. **c**. Shows the same samples as in a) developed with anti-mouse kappa demonstrating that the IgM was intact as both heavy and light chains are detected in the pentameric molecular weight range. The pooled results are shown on Figure 4b blots and graphs.

.
